# Gut microbial dataset of a foraging tribe from rural West Bengal - insights into unadulterated and transitional microbial abundance

**DOI:** 10.1016/j.dib.2019.103963

**Published:** 2019-05-24

**Authors:** Sayak Ganguli, Somosree Pal, Kaustav Das, Rajat Banerjee, Subrata Sankar Bagchi

**Affiliations:** a Theoretical and Computational Biology Unit, AIIST - Palta and the Biome, Kolkata 700064, India; b Department of Anthropology, Bangabasi College, Kolkata 700019, India; c Department of Biotechnology, University of Calcutta, Kolkata 700019, India

**Keywords:** Savars, Gut microbial profiles, Transitional microbiome

## Abstract

The human gut microbiome contributes to a broad range of biochemical and metabolic functions that directly or indirectly affect human system. Numerous factors such as age, geographical location, genetic makeup, and individual health status significantly influence the diversity, stability, and relative abundance of the gut microbiome. Of the mentioned factors, geographical location and dietary practices appears to explain a significant portion of microbiome variation. On the other hand tribal people living in geographically isolated areas and dependent on their traditional food sources are considered as having relatively unadulterated gut as their guts are least colonized by Western diet. The Western diet — low in fiber and high in refined sugars — is basically wiping out species of bacteria from our intestines. That's the conclusion Smits (2017) and his team reached after analyzing the Hadza microbiome at one stage of their year long study. The trend was clear: The further away people's diets are from a Western diet, the greater the variety of microbes they tend to have in their guts. And that includes bacteria that are missing from American guts."So whether it's people in Africa, Papua New Guinea or South America, communities that live a traditional lifestyle have common gut microbes — ones that we all lack in the industrialized world. In this work we present a pilot study data of the gut microbiome of an ethnic tribe of West Bengal, India, originating from Dravidian descent - the Savars. These are nomadic tribes and are still dependent on hunting and gathering for their livelihood. We identified a healthy family and have analysed their stool samples for gut microbial profiles.

**Table:** Specifications table

Subject area	*Biology*
More specific subject area	*Gut Microbial Profiling*
Type of data	*NGS Based Data represented in form of Pie chart and Heat Map*
How data was acquired	*Illumina Hiseq Next Gen Sequencing Platform; FASTQC, QIIME*
Data format	*Raw FASTQ files*
Experimental factors	Subjects were allowed to have their regular diet
Experimental features	*DNA isolation and sequencing from first faecal matter of the subjects*
Data source location	Bangabasi College, Kolkata
Data accessibility	https://www.ncbi.nlm.nih.gov/sra/SRX5459403[accn] - SRA https://www.ncbi.nlm.nih.gov/bioproject/525343 - Bioproject
Related research article	Smits, S. A., Leach, J., Sonnenburg, E. D., Gonzalez, C. G., Lichtman, J. S., Reid, G., Knight, R., Manjurano, A., Changalucha, J., Elias, J. E., Dominguez-Bello, M. G., … Sonnenburg, J. L. (2017). Seasonal cycling in the gut microbiome of the Hadza hunter-gatherers of Tanzania. Science (New York, N.Y.), 357(6353), 802–806 [1].

**Table:** 

**Value of the data** • The significance of this data stems from the fact that this is the first report of the gut microbial abundance from SAVARS of West Bengal, a nomadic tribe who are still basically foragers i.e. essentially carnivores and dependent on forest produce, having minimum or no direct agricultural practice. Thus future studies which plan to incorporate comparisons of urban and rural gut microbiomes can use this a reference point to compare and reveal the commonalities and variations that have been induced in the gut as a result of the socioeconomic lifestyle patterns. • The gut microbial profiles identified represent unadulterated gut which has not been influenced by fast food and ready to cook frozen and stored commercial products. Further, these guts still are shielded from the overuse of medicines and antibiotics, thus antibiotic resistant microbes should be a rarity in the profiles. • The data also reveals the propensity of transition of the gut microbial profiles from father to son and mother to son thus revealing the parental contribution to the formation of the child's gut microbiome.

## 1 Data

**Fig. 1:**
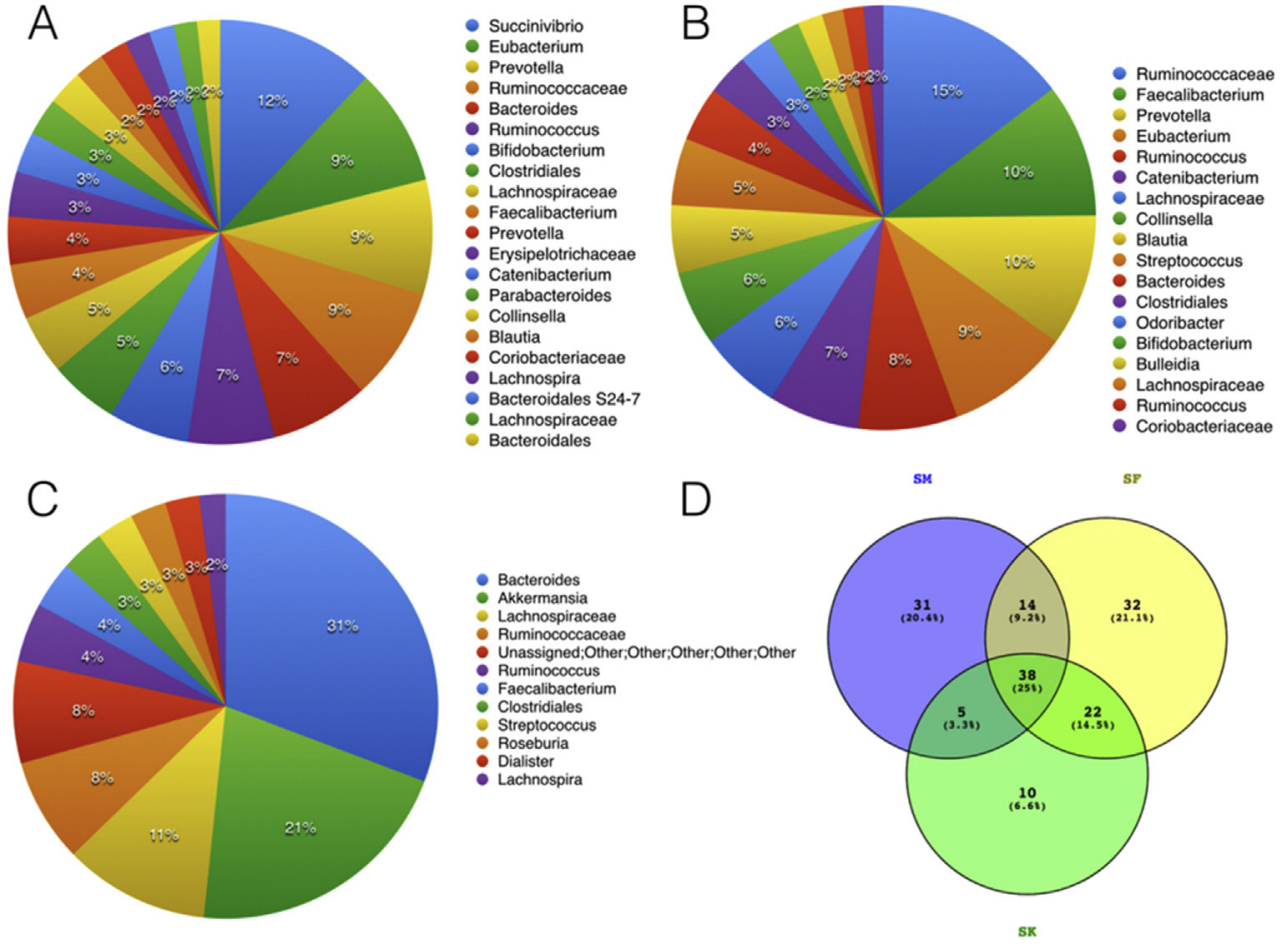
Gut Microbial Profile of the Savar family under study. A Male; B: Female and C: Child (male); D: Common taxa between the members; where SM = Male; SF=Female and SK=Child.

This data describes the gut microbiome profile of a nomadic tribe of west bengal - “Savar”. This tribe has till date not adapted to the agricultural practices and is still dependent on hunting and forest produce for their livelihood, making their gut a quintessential reservoir for unadulterated microbes. Three datasets are presented - one belonging to a SAVAR male (Age: 30 years), the other of a female (Age: 26 years) and the third of their male child (Age: 7 years) who is yet to attain puberty. Ruminococcaceae (15%); Succinovibrio(12%) and Bacterioides(31%) were identified to be the most abundant of the microbial communities in female, male and child respectively (Fig. 1).

## 2 Experimental design, materials, and methods

The collection of data was performed in two important steps:

a) Counselling and Medical Evaluation of the Subjects:

The subjects were initially counselled on the requirement of the first faecal matter and were allowed to feed on their regular diet comprising of staple rice, poultry meat, fish and forest produce such as fruits for a week. Basic health check up of blood pressure and BMI (Body Mass Index) were performed to ensure that they were healthy, with no previous history of ailment or chronic infection in the last six months prior to sample collection with the help of a skilled medical professional.

**Fig. 2:**
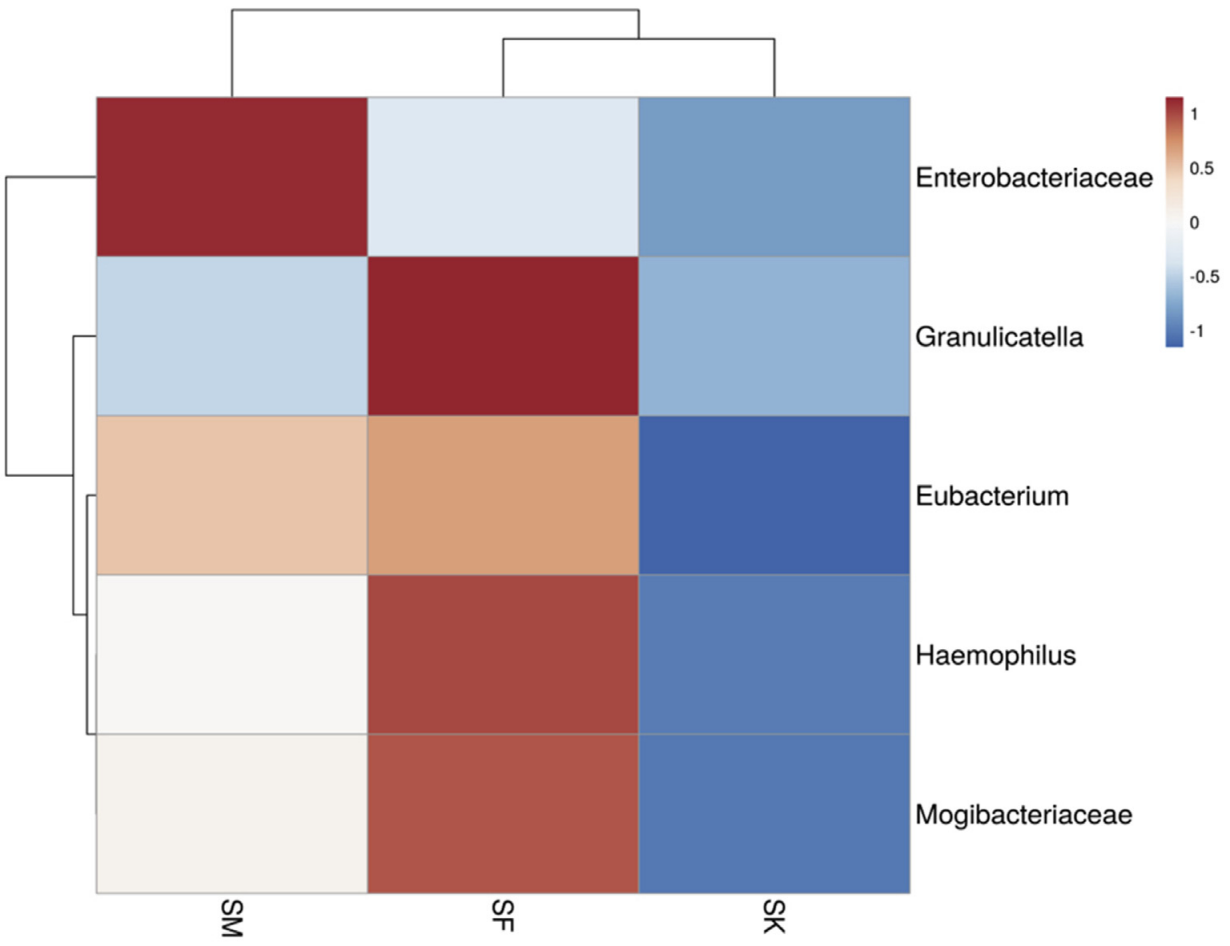
Heatmap representing the top common taxa between the members under study, where SM = Male; SF=Female and SK=Child.

b) Collection of First Faecal Matter and Sequencing:

The first faecal matter was collected at 5.30am in the morning in sterile containers previously autoclaved. The medical profiling of the candidates were again performed following the procedure and all of the subjects. The faecal matter was packed in containers and sealed with paraffin and transported to the sequencing facility within 10 hours of collection. Sequencing was performed using Illumina chemistry in the Hiseq platform using the protocol described in Bag et al. [2]. with the Bioinformatics pipeline as previously described [3]. Following this, standard bioinformatics pipeline utilizing FASTQC, QIIME [4], and Krona, SILVA [5] and Greengenes [6] databases were used to first quality check the data and then identify the taxa. Following this the common taxa between the subjects [male vs female; male vs kid and female vs kid; Fig. 1D] were identified and the most abundant of the members were analysed using a heat-map [Fig. 2].

## References

[1] Smits S.A., Leach J., Sonnenburg E.D., Gonzalez C.G., Lichtman J.S., Reid G., Knight R., Manjurano A., Changalucha J., Elias J.E., Dominguez-Bello M.G., Sonnenburg J.L. (2017). Seasonal cycling in the gut microbiome of the Hadza hunter-gatherers of Tanzania. Science.

[2] Bag, Satyabrata, Saha, Bipasa,Mehta, Ojasvi, Anbumani, D…..Das, Bhabatosh; An improved method for high quality metagenomics DNA extraction from human and environmental samples; Sci. Rep., 10.1038/srep26775%2010.1038/srep26775. PMC4886217 27240745

[3] Ganguli S., Rahaman S., Bera A.R., Vishal V., Malik S., Roopalakshmi K., Singh P.K. (2017). Rhizospheric metagenome of the terrestrial mangrove fern *Acrostichum* from Indian Sunderbans. Genomics Data.

[4] Caporaso J Gregory, Kuczynski Justin, Stombaugh Jesse, Bittinger Kyle, Bushman Frederic D., Costello Elizabeth K., Fierer Noah, Gonzalez Pena Antonio, Goodrich Julia K., Gordon Jeffrey I., Huttley Gavin A., Kelley Scott T., Knights Dan, Koenig Jeremy E., Ley Ruth E., Lozupone Catherine A., McDonald Daniel, Muegge Brian D., Pirrung Meg, Reeder Jens, Sevinsky Joel R., Turnbaugh Peter J., Walters William A, Widmann Jeremy, Yatsunenko Tanya, Zaneveld Jesse, Knight Rob (2010). QIIME allows analysis of high-throughput community sequencing data. Nat. Methods.

[5] Quast C., Pruesse E., Yilmaz P., Gerken J., Schweer T., Yarza P., Peplies J., Glöckner F.O. (2013). The SILVA ribosomal RNA gene database project: improved data processing and web-based tools. Opens external link in new windowNucl. Acids Res.

[6] DeSantis T.Z., Hugenholtz P., Larsen N., Rojas M., Brodie E.L., Keller K., Huber T., Dalevi D., Hu P., Andersen G.L. (2006). Greengenes, a chimera-checked 16S rRNA gene database and workbench compatible with ARBAppl. Environ. Microbiol. Jul.

